# The Dangers of Arsenic

**Published:** 1902-05-03

**Authors:** 


### THE DANGERS OF ARSENIC.

In the course of one of the usual afternoon consultations at the Polyclinic, Mr. Jonathan Hutchinson lately had a case which illustrated the risks which sometimes attend the administration of arsenic, and served well to show how necessary it is to take care not to carry the use of this drug too far. The patient had been ordered arsenic seven years before, for the treatment of lupus erythematosus, and had continued it ever since! So far as the lupus was concerned it had done well, for this was cured, but it had caused great discoloration of the skin generally and had also set up a condition of the palms of the hands and the soles of the feet to which Mr. Hutchinson specially directed attention. In these parts the skin was roughened, not all over but in patches, little horny patches of heaped up and adherent epidermis, somewhat of the nature of corns, being scattered over the palmar surface. A distinction was to be drawn between warty and "corny" growths. A wart was a papillary outgrowth, a corn was an epidermic growth, and although when a corn produced irritation the papillæ were no doubt affected, the growth itself was purely epidermic. This was the case in the patient who was the subject of these remarks. Turning from the particular case to the general question, Mr. Hutchinson said that it must always be remembered in regard to arsenic, that it was a profound modifier of nutrition, and that although in its action on the skin its first effect was to render it clear and "transparent," the result of its prolonged or excessive use was to produce "muddiness" and discolouration, and ultimately an epidermic overgrowth in parts. The case before them was a good example of this; the mottled discolouration of the skin of the face, the neck, and the upper part of the chest, which they were all able to see, showed well the nature of the pigmentation produced, and he could tell them that in some other parts of the body the skin was almost black. As to the condition of the palms, that was a state of affairs which, although but recently observed, was now well recognised as being the result of arsenic poisoning, and he was able by means of drawings from the Museum to show other examples of its occurrence in this connection. The moral of the case then was very definite, namely, that during the administration of arsenic the skin must be carefully watched, and that when any sign of discolouration appeared the drug must be diminished if not stopped. When, however, any sign of epidermic overgrowth appeared it was urgently necessary that the drug should be stopped, absolutely and immediately, and this for a reason which he had not mentioned while the patient was in the room, namely, that these horny growths might, and sometimes did, lead to epithelioma. Of this he had seen many instances, and, again drawing upon the pictures in the Museum, he gave examples illustrating this unfortunate sequence of events. No doubt the form of epithelioma which occurred in connection with the administration of arsenic differed somewhat from the forms of the disease which were most commonly met with, but, as in one of the cases which he more fully described, there could be no doubt whatever of its cancerous nature, ending as it did with secondary deposits in the glands and on the ribs and in other parts. Not only, said Mr. Hutchinson, was it an important matter in regard to the adminstration of arsenic to remember that if carried too far it might lead to this disease, but from a pathological and etiological standpoint it was a matter of no small interest to see that the administration of a drug—a mere chemical poison like arsenic—could so profoundly modify nutrition as to lead to the development of cancer. A very interesting matter in regard to the cancerous growths developed under these conditions was that they were apt to be multiple, the primary disease occurring in several separate foci. Mr. Hutchinson added that, without saying anything as to what might turn out to be the actual cause of cancer, the fact that by the administration of a chemical poison so great a tendency to it could be set up as to cause the disease to break out in several distinct parts of the body, went far to suggest that the real origin of cancer was to be sought in some modification of nutrition rather than in that intrusion of infective agents towards which many pathologists had of late shown so great a leaning.

